# Trusted authorities can change minds and shift norms during conflict

**DOI:** 10.1073/pnas.2105570118

**Published:** 2021-10-11

**Authors:** Graeme Blair, Rebecca Littman, Elizabeth R. Nugent, Rebecca Wolfe, Mohammed Bukar, Benjamin Crisman, Anthony Etim, Chad Hazlett, Jiyoung Kim

**Affiliations:** ^a^Department of Political Science, University of California, Los Angeles, CA 90095;; ^b^Department of Psychology, University of Illinois Chicago, Chicago, IL 60607;; ^c^Department of Political Science, Yale University, New Haven, CT 06520;; ^d^Harris School for Public Policy, University of Chicago, Chicago, IL 60637;; ^e^Mobukar Research Consultancy Services, Ltd., 600222 Maiduguri, Nigeria;; ^f^Department of Politics, Princeton University, Princeton, NJ 08544;; ^g^Mercy Corps, 900108 Maiduguri, Nigeria;; ^h^Department of Statistics, University of California, Los Angeles, CA 90095

**Keywords:** violent extremism, leaders, conflict, norms, reintegration

## Abstract

The reintegration of former members of violent extremist groups is a pressing policy challenge. Governments and policymakers often have to change minds among reticent populations and shift perceived community norms in order to pave the way for peaceful reintegration. How can they do so on a mass scale? Previous research shows that messages from trusted authorities can be effective in creating attitude change and shifting perceptions of social norms. In this study, we test whether messages from religious leaders—trusted authorities in many communities worldwide—can change minds and shift norms around an issue related to conflict resolution: the reintegration of former members of violent extremist groups. Our study takes place in Maiduguri, Nigeria, the birthplace of the violent extremist group Boko Haram. Participants were randomly assigned to listen to either a placebo radio message or to a treatment message from a religious leader emphasizing the importance of forgiveness, announcing the leader’s forgiveness of repentant fighters, and calling on followers to forgive. Participants were then asked about their attitudes, intended behaviors, and perceptions of social norms surrounding the reintegration of an ex–Boko Haram fighter. The religious leader message significantly increased support for reintegration and willingness to interact with the ex-fighter in social, political, and economic life (8 to 10 percentage points). It also shifted people’s beliefs that others in their community were more supportive of reintegration (6 to 10 percentage points). Our findings suggest that trusted authorities such as religious leaders can be effective messengers for promoting peace.

## Significance

Violent extremist groups such as the Islamic State and Boko Haram have proliferated across the world in recent decades. While considerable scholarship addresses why people join violent extremist groups, much less attention has been paid to how former members reenter society. Yet successfully ending conflict requires reluctant communities to accept former members back home. In this research, we find that radio messages delivered by trusted authorities in Nigeria lead to large, positive changes in people’s willingness to accept former Boko Haram fighters back home and make people think their neighbors are more in favor of reintegration. Our results show that messages from leaders can create change on a mass scale at low cost, helping to end conflict and division.

Violent extremist groups such as al-Qaida, al-Shabab, Boko Haram, and the Islamic State and its regional affiliates have proliferated across the world in recent decades, killing hundreds of thousands and displacing millions more (1, 2). Considerable recent scholarship addresses the question of who joins violent extremist groups and what motivates people to join (e.g., refs. 3–11). Less attention has been paid to the systematic study of how former members of violent extremist groups reenter society (but see ref. 12), despite the fact that this has become an urgent and growing policy challenge. In the wake of conflicts in Iraq, Nigeria, Syria, the United States, Europe, and beyond, former members of violent extremist groups are attempting to reintegrate into society through informal as well as formal, government-led processes (13–17).

While former combatants have generally been welcomed back home in civil war contexts after intensive reintegration campaigns (18, 19), the reintegration of former violent extremists has proved more challenging (12, 13, 20, 21). Violent extremist groups not only use violent tactics targeted at civilians, such as suicide bombings and armed attacks, but also indoctrinate members into extremist ideologies. As a result, policymakers and citizens are often afraid that defectors still believe in extremist ideology and may be motivated to carry out violence back at home or recruit others into the group.

Yet putting an end to conflict and preventing the continuation of cycles of violence requires finding ways for former members of extremist groups to rejoin social, political, and economic life at home (22). Former violent extremists with nowhere to go may rejoin their extremist groups or form new ones. Moreover, the lack of a viable path out of a violent extremist group and back into civilian life may dissuade current fighters from defecting (23).

Creating the conditions for peaceful reintegration of former fighters requires change on a mass scale in advance of their return. Acceptance is a collective process requiring the assent of many, such as government officials, community leaders, family members, future neighbors, and local business owners (24). While the ultimate goal is to change behaviors to foster reintegration, governments and civil society must first address the reticence of communities to accept ex-fighters back home (15). This process starts by changing community members’ attitudes, intended behaviors, and perceived community norms (i.e., perceptions of what others in one’s community think; refs. 25, 26) around reintegration. If most citizens are personally supportive of reintegration, intend to accept returning fighters, and believe others in their community are also supportive of reintegration, this will signal to government officials that reintegration efforts can begin, help prevent conflict when former combatants return home, and encourage the demobilization and return of former fighters ready to lay down arms.

In this paper, we examine a highly scalable intervention that we hypothesize will be effective at changing minds and shifting norms surrounding the acceptance of former fighters: having trusted authorities spread messages in support of reintegration. This intervention is promising because it has the potential to create change on a mass scale by spreading messages in the media and at large public gatherings. Converging evidence from political science and psychology on the effects of cues from elites, moral authorities, and group leaders suggests that messages from trusted authorities can be effective in both changing people’s attitudes as well as shifting social norms. People often form beliefs and make decisions based on cues from their leaders (27–29). Additionally, leaders’ words and actions serve as signals of social norms: people assume that others in their community will observe and be influenced by their leader’s messages or actions, which in turn can shift people’s own attitudes and behaviors (30–32).

Moreover, messages from trusted authorities are commonly employed by policymakers to combat violent extremism (33). However, little previous research examines whether such messages are effective in changing attitudes and norms around the challenges in ending violent conflict. Indeed, one recent study in Burkina Faso finds that messages from religious leaders are not effective in changing attitudes on violent extremism (34), raising the question of whether this type of intervention will be effective when it comes to reintegration. Yet, encouragingly, recent research in civil war contexts in West Africa suggests that authorities, such as local leaders and international peacekeepers, can play important roles in preventing or resolving conflict (35–37).

## Context

In the present study, we examine whether messages from trusted authorities can change people’s attitudes, intended behaviors, and perceptions of community norms around the reintegration of former members of the Salafi-Jihadist group Boko Haram in northeastern Nigeria. Boko Haram promotes an extremist form of Islam and targets both Muslim and non-Muslim communities. The conflict, which started in 2009, has killed tens of thousands and displaced millions (38). While the conflict continues unabated, thousands of male fighters—together with men, women, and children who played support roles—have escaped, surrendered, or been captured. Many are now entering a government-led detention and rehabilitation process for repentant former Boko Haram members or are attempting to informally reintegrate into their home communities (15).

However, those seeking to reintegrate face a suspicious, sometimes hostile, population (39). Forty percent of people in our placebo-control group reported that they are unwilling to accept a former Boko Haram fighter back into their community, and 43% think that former Boko Haram members will try to harm people rather than live peacefully in the community.

Successfully ending this conflict will require these reluctant and fearful communities to accept former fighters and affiliates back home as neighbors. Finding ways for governments or other organizations to address community concerns and ultimately change the way communities feel about reintegration is therefore paramount to preventing violence.

## Religious Leaders as Trusted Authorities

To develop the intervention in northeastern Nigeria, we first identified what kind of leaders are most trusted by the general population. In qualitative interviews and focus groups conducted prior to our experimental study, many people expressed high levels of trust in and deference toward religious authorities, from their local imams to national religious leaders, on a number of issues, including reintegration. This is not surprising, given that religion plays an important role in daily life in northeastern Nigeria. In our placebo-control group, 97% of people report trusting their religious leaders. Moreover, at the time of our study, a number of religious leaders were already involved in efforts to prepare communities for reintegration by including messages about the importance of forgiveness in their sermons and on radio programs.

We note that northeastern Nigeria is not alone in its high levels of trust and deference to religious authorities. The vast majority of the world’s population is highly religious, particularly in the Global South (40). In addition to being trusted authorities in many communities worldwide, religious leaders have the authority to advise their followers on a variety of topics, such as politics, economics, justice, and health, by drawing on religious tenets and texts with legitimacy (41). Messages from religious leaders on such topics reach billions of people around the world each week.

Previous research on the influence of religious leaders relies largely on evidence from observational studies. A small but growing number of experimental studies focus on the effects of presenting people with the content of religious texts, delivered without attribution to a religious authority (42–44) or with attribution (45). The effects of these texts likely differ when incorporated into sermons and private counsel delivered by religious leaders.

## Methods

We designed an experiment in cooperation with Mercy Corps, an international humanitarian and development organization working in northeastern Nigeria, to test whether messages from religious leaders can influence attitudes and intended behaviors, as well as shift perceived social norms, related to community support for the reintegration of former Boko Haram members. The experiment was conducted from August to October 2018 with a representative sample (*n* = 1,452) of adult Muslim residents in Maiduguri, Nigeria, the birthplace of Boko Haram.* Participants were recruited from 67 urban and semiurban neighborhoods and camps for internally displaced persons (IDPs) and were interviewed face-to-face in their communities. They were randomly assigned to listen to either a treatment message from a religious leader (*n* = 699) or to a placebo radio message on a topic unrelated to the Boko Haram conflict (*n* = 753). Power calculations were conducted using DeclareDesign (46).

Study communities were randomly selected from a list of all neighborhoods and IDP camps in the Maiduguri metropolitan area that passed an initial security check. Within each community, we used a random walk procedure to select households and then randomly selected one adult from each household to participate in the study. Interviews were conducted using Qualtrics Offline software on smartphones by a team of 25 local researchers who were all from Maiduguri town and were trained over a 2-wk period by the authors. Interviewers were matched to the respondent’s language and gender.

### Developing the Messages.

We developed the religious leader message in collaboration with a senior Islamic sheik, who recorded the message in his own voice in both Hausa and Kanuri, the two most common languages in Maiduguri. The message content was pilot-tested using focus groups and individual interviews. The message began with an announcer welcoming everyone to today’s radio program and explaining that listeners would hear from “Goni Muhammad Sa’ad Ngamdu himself, a known Muslim cleric in Borno State.” The Sheikh then delivered his message about reintegration. The message had three key elements: 1) emphasizing the importance of forgiveness in religious texts, 2) announcing that the leader would forgive repentant former Boko Haram members, and 3) calling on followers to forgive as well. The announcer then closed the program by thanking everyone for listening.

The placebo radio message was designed to control for the experience of listening to a radio message in the middle of the study. The same announcer opened the program, and the intro and outro text was as similar as possible to the treatment condition. The announcer then transitioned to the day’s guest, who would provide information on how to stay healthy by using safe water, sanitation, and hygiene practices (the study was conducted before the COVID-19 pandemic). The placebo message was not delivered by a religious leader. The full text of the treatment and placebo messages can be found in *SI Appendix*, Tables S1 and S2.

### Procedure and Measurement.

Once an individual was randomly selected from within their household to participate in the study, they went through a detailed informed consent procedure. They were told that the survey would ask about their experiences with the Boko Haram conflict, including questions on their opinion of people who were associated with Boko Haram. The interviewer explained that they were working with an international nongovernmental organization and an American university. They also emphasized that they were in the community to do research and that the survey questions would not have anything to do with the distribution of aid. Respondents were not offered any compensation for their participation in the survey due to the policies of our partner organization and were informed of this.

If an individual consented to participate in the study, the interviewer started by asking a number of pretreatment questions on demographics, experiences with Boko Haram violence, and mental health. After this series of questions, the interviewer explained that they had been asking a lot of questions, so now they were going to do something different and play a clip from a radio program. Since people in northeastern Nigeria typically listen to the radio communally, we incorporated a communal listening experience into the study design in order to make the treatment more naturalistic. We did so for generalizability reasons: listening to a radio program alone likely has a different effect than listening in a group. When listening in a group, people can observe how others react verbally and nonverbally to the content, which in turn could influence their perceptions of social norms and their own attitudes. To create a group experience, the interviewer asked the participant to gather a few of their family members or neighbors to join for the radio program.

Once the participant gathered their family members or neighbors, the interviewer connected the smartphone to a speaker and played one of the two audio messages to the small group. The treatment condition was randomly assigned from within the Qualtrics Offline survey, using simple random assignment with equal probabilities across participants. The interviewer did not know which program would air until they pressed play on their survey.

When the program was over, the interviewer thanked the others for joining and politely asked them to leave in order to continue the interview with the respondent in private. Next, participants completed an immersive distractor task. They were then given a short vignette about a hypothetical ex–Boko Haram fighter. Characteristics of the ex-fighter’s profile were randomly assigned, such as his age, length of time in Boko Haram, and whether he is described as repentant (Table 1). This was done in order to ensure that effects were not dependent on particular characteristics of the hypothetical ex-fighter.

**Table 1.** Hypothetical Boko Haram fighter vignette

I want to tell you the story of *[ Ali / Usman ]* a *[16 / 21 / 26 / 31]* year old man from your community. Before the crisis, *[ Ali / Usman ] [ was living with his parents. In the morning, he swept the front of his house. Most evenings, he played football on a field in the community. / was a small trader selling vegetables before the crisis. He had two younger sisters. In the morning, he went to the market and then sold on a street in his community. ]* When Boko Haram came to the community, he was convinced by their preaching and willingly decided to join them. He was with them for *[ 1 mo / 1 y / 2 y ]*, and his role was mostly as a fighter. *[ He escaped their camp one afternoon, and walked until he reached a nearby town. / He was captured by the military. / He surrendered during a battle. ] [ He has been released by the government after rehabilitation. / No mention.]* *[ He is willing to swear on the Koran to show the community that he is repentant and will never go back to Boko Haram. / He is willing to swear on the Koran to show the community that he is repentant. / No mention.]*

The vignette was read to respondents before asking outcome questions regarding this hypothetical former Boko Haram fighter. Italicized text was randomized independent of the main treatment.

Participants were then asked an outcome question measuring attitudes (specifically, “If the decision was yours alone, do you think he should be allowed to stay in the community?”), several intended behaviors (e.g., “Would you trade with him?”), and perceptions of social norms about the willingness of their neighbors, community leader, and local religious leader to accept the ex-fighter back into the community (e.g., “Now I want you to think about the rest of the people in your community. Do you think they would agree for him to stay in the community?”). We average the six behavioral intention items to create the “Willingness to Interact Index” and average the three perceived social norms items into the “Perceived Norms Index.” The indices are constructed by taking the mean across all items in the index that a respondent answered (except for attitudes, which are measured using a single item). We also measure the emotions of anger at the ex-fighter for what he has done in the past and fear of what the ex-fighter might do in the future. The full text of the outcome measures can be found in *SI Appendix*, Table S3. All survey questions were translated into Hausa and Kanuri. The questions were also pilot-tested to make sure they were appropriate for the local context and that the translation into both languages correctly captured the meaning of each question consistently.

### Safety and Ethics.

Conducting a study in an active conflict zone raises a number of safety and ethical concerns that must be addressed. Our main priority was to ensure that we never put either interviewers or respondents at risk during the data collection. We addressed this challenge in a number of ways. First, we worked closely with the security team from our partner organization, Mercy Corps. We checked in with them each day to make sure that it was safe to travel to the day’s selected community. Second, we contacted community leaders in advance of our visit to get permission to come into their community and conduct the study. We would also visit the community leader upon entry into the community, and the leader would typically assign a few respected community members to accompany our team. Third, we made sure to finish all of our interviews in each community within 1 d. Finally, the interviewers were instructed to stop an interview or leave a community immediately if they felt any threat to their safety.

Another priority was to make sure that we mitigated negative consequences to respondents and interviewers that may arise from participating in a survey that included detailed questions on violence and trauma. Interviewers went through 2 wk of training before the survey. The training included mock interviews in which we acted out difficult scenarios that may arise in the field and discussed techniques to deal with upset or traumatized respondents. To address possible negative consequences for respondents, everyone who participated in the survey received a psychosocial referral card at the end of the interview. The card had information about the psychosocial counseling network in Maiduguri, which is accessible to anyone for free. Interviewers also offered to assist respondents if they needed help navigating the psychosocial referral process. The study was approved by the University of California, Los Angeles institutional review board.

### Analysis.

We estimate treatment effects and calculate adjusted conditional means using a linear regression model to adjust for the characteristics of the hypothetical fighter that varied in the vignettes. We use demeaned indicators for each vignette feature, interacted with the treatment, to improve efficiency (47).^†^ We report heteroskedasticity-consistent SEs (specifically the “HC2” estimator). The study hypotheses and analyses were preregistered on the Open Science Framework (OSF) at https://osf.io/pu5yr/.

## Descriptive Statistics

Our sample is 57% female, with an average age of 35 (median = 32; range = 18 to 90). Forty-nine percent have no formal education, and 29% are completely unemployed, meaning they have no formal or informal work. The sample is also highly war affected: 63% were displaced at some point during the conflict, including 41% who were displaced at the time of the survey. Experiences with violence at the hands of Boko Haram are extremely common: 47% of respondents had their property destroyed by Boko Haram, 26% witnessed Boko Haram killing someone, and 14% were personally beaten or tortured. Sixty-seven percent had a relative killed by Boko Haram, and 31% had a relative abducted. Not surprisingly, respondents report experiencing many symptoms associated with posttraumatic stress disorder. For example, 46% experience nightmares and 36% have flashbacks.

In terms of attitudes toward reintegration, 40% of respondents in the placebo-control condition say that the ex-fighter featured in the vignette should not be allowed to return to their community. Forty-two percent would not allow the former fighter to attend community meetings, and 46% would not trade with him in the market. Thirty-six percent of placebo-control respondents report feeling angry at the ex-fighter for what he has done in the past, and 53% are afraid of what he might do in the future. With respect to community norms around reintegration, 28% of respondents say that none of their neighbors would be willing to accept the former fighter back into the community, and 48% say that only some of their neighbors would be willing to accept. Thirty-nine percent think that their local community leader would not allow the ex-fighter to return, and 39% think their local religious leader would not allow him to return.

## Results

**Fig. 1.**
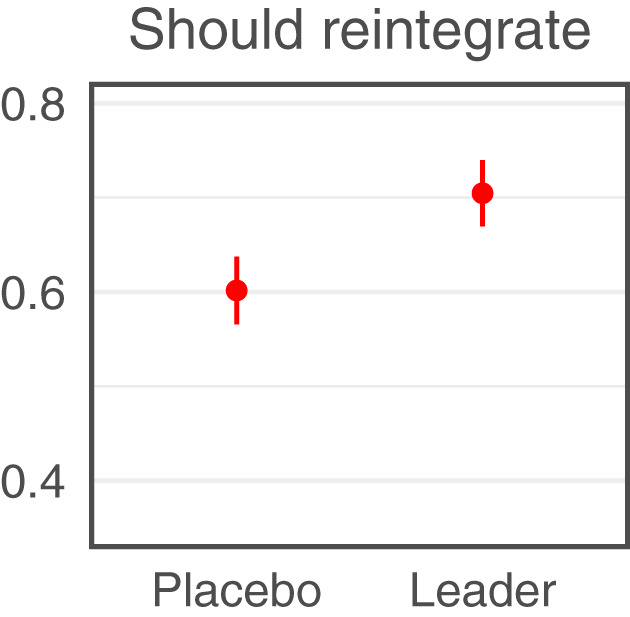
Messages from religious leaders change attitudes toward reintegration. We present regression-adjusted conditional means (red dot) with 95% CIs (red lines) for the placebo-control group (“Placebo”) and the religious leader treatment group (“Leader”) for attitudes toward reintegration. The vertical axis represents the proportion of “yes” responses.

**Fig. 2.**
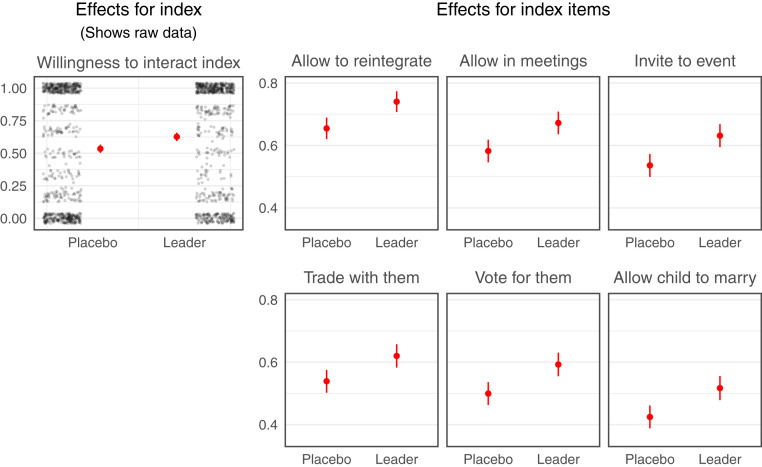
Messages from religious leaders change willingness to interact. We present raw outcome data (jittered black dots) and regression-adjusted conditional means (red dots) with 95% CIs (red lines) for the placebo-control group (“Placebo”) and the religious leader treatment group (“Leader”) for the “Willingness to Interact Index.” We also present regression-adjusted conditional means and 95% CIs for the six constituent items. The vertical axis for each item represents the proportion of “yes” responses; the vertical axis for the index plot represents points on that index, which is an average of the proportion of “yes” responses on the component items.

We find that listening to a message from a religious leader changes people’s minds about reintegration, positively shifting both people’s attitudes and behavioral intentions toward accepting former fighters. In terms of attitudes, 60% of respondents in the placebo group say that they think former members should be allowed to reintegrate into their community (SD = 49); this number increases by 10 percentage points for those who listen to the religious leader message (95% CI: 5 to 15; *P* = 0.00006; Fig. 1; regression estimates in *SI Appendix*, Table S4). In terms of behavioral intentions, the increase is 9 points on the “Willingness to Interact Index” (95% CI: 5 to 14; *P* = 0.00007). This is a substantial effect, given that the mean in the placebo group is 53% (SD = 44). Effects range from 8 to 10 percentage points across index items (Fig. 2; regression estimates in *SI Appendix*, Table S5). For example, 53% of respondents in the placebo group say that they would allow former members to attend their wedding or their child’s naming ceremony; this number increases by 10 percentage points for those who listen to the religious leader message (95% CI: 4 to 15; *P* = 0.0003).

**Fig. 3.**
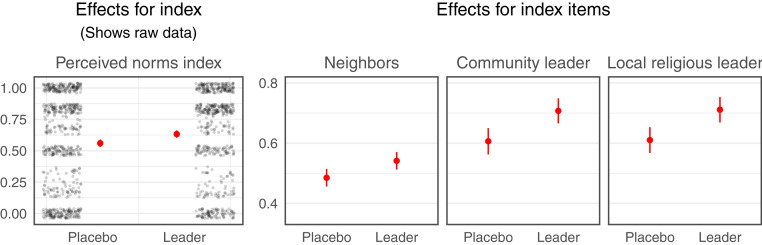
Messages from religious leaders shift perceived norms. We present raw outcome data (jittered black dots) and regression-adjusted conditional means (red dots) with 95% CIs (red lines) for the placebo-control group (“Placebo”) and the religious leader treatment group (“Leader”) for the “Perceived Norms Index.” We also present regression-adjusted conditional means and 95% CIs for the three constituent items. The vertical axis for each item represents the proportion of "yes" responses; the vertical axis for the index plot represents points on that index, which is an average of the proportion of "yes" responses on the component items.

The religious leader message also positively shifts people’s perceptions of social norms around reintegration in their community (placebo group mean = 56%; SD = 38). Participants who listen to the treatment (versus placebo) message report thinking that their neighbors, community leader, and local religious leader are more supportive of reintegration (average effect on the “Perceived Norms Index”: 7 points, 95% CI: 3 to 11, *P* = 0.0005; Fig. 3; regression estimates in *SI Appendix*, Table S6). Effects range from 6 to 10 percentage points across items.

These effects are not moderated by any of the randomly varied characteristics of the hypothetical ex-fighter profile. The only characteristic that influenced mean levels of acceptance in the main indices was whether or not the ex-fighter was described as repentant. The Perceived Norms Index was also influenced by the age of the hypothetical former fighter. We also find little heterogeneity in effects for any of the main outcomes according to any of the respondent characteristics we preregistered, including measures of religiosity, trust in political and religious leaders, and authoritarian personality (*SI Appendix*, Tables S12–S14).

The religious leader message also shifts attitudes, intended behaviors, and norms toward two additional hypothetical Boko Haram members: a man and a woman described as being abducted into the group. The effects are generally positive, large, and statistically significant (*SI Appendix*, Tables S15–S18).

However, we do not see effects on anger and fear toward former Boko Haram members (anger: −2 points, 95% CI: −7 to 4, *P* = 0.56; fear: −0.007 points, 95% CI: −6 to 5, *P* = 0.78; *SI Appendix*, Table S19). This suggests that the patterns we observe are not simply a result of demand effects, as participants are not responding positively to all outcome questions as a result of hearing a message in favor of reintegration. It also raises an interesting question about why the intervention improves reintegration attitudes but does not reduce anger and fear. A likely explanation is that the message content is designed more to influence how people think about reintegration rather than how they feel about former Boko Haram affiliates. It provides information about what Islamic texts say about forgiveness, informs listeners that their religious leader is in favor of reintegration, and includes an appeal from the leader for them to support reintegration as well. Perhaps the message would need a more explicit appeal targeted at emotions in order to reduce anger and fear.

## Discussion

Our findings provide experimental evidence that messages from religious leaders can shift personal attitudes, intended behaviors, and perceived social norms around the reintegration of former members of a violent extremist group. After the intervention, people are more likely to say they will allow former members of Boko Haram to rejoin the community and participate actively in social, economic, and political life. They are also more likely to believe that their neighbors and local leaders are supportive of former members returning. These results are encouraging, because if even a small number of ex-members are reluctant to reintegrate due to fears that their communities won’t accept them, the risks of instability remain. We consider this study to be a first step in a broader research agenda investigating whether religious authorities are effective messengers in conflict contexts and should be centrally involved in conflict resolution. In that spirit, we note here some limitations for our study that may inspire future research.

First, we measure the effect of the religious leader message on attitudes, perceived norms, and intended behaviors, not on actual behaviors. While our goal for this particular study was to test whether messages from trusted authorities could change minds and shift norms in order to pave the way for peaceful reintegration, the question remains whether such messages would be effective at changing behaviors down the line and how long that effect might last. While behavioral intentions do not always predict future behavior, perceived social norms often do (32, 48). Given this, as well as the brevity of our intervention and the magnitude of observed effects on attitudes, norms, and intended behaviors, we think there is reason for optimism that trusted authorities will also influence future reintegration behaviors. Additionally, we measure effects immediately after listening to the radio program. Due to respondent safety concerns, we were not able to collect follow-up data to examine whether the effects persist. Previous research finds mixed evidence as to whether a brief messaging or media intervention leads to lasting change (49–51). It is possible—and perhaps even likely—that listening to one relatively short message from a religious leader may not lead to enduring effects. However, hearing repeated messages from religious leaders on the radio or in the mosque might be more likely to create lasting change.

Second, we measure the effect of a message delivered by a religious leader. The use of religious leaders makes sense in our study’s context: northeastern Nigeria is a highly religious society, making religious authorities plausibly influential. Moreover, religion is at the center of the Boko Haram conflict that defines this context. Beyond Nigeria, we expect that religious leaders will be effective at influencing attitudes, norms, and behaviors among religious populations. Most religions lay out many prescriptions relevant to issues of politics, economics, justice, and social organization (52), and thus religious leaders can and do credibly speak about and likely influence people in a number of domains (45, 53–55). Additionally, while our study had a message from a high-profile religious authority, we would expect messages from more local-level religious authorities to be similarly effective. While local leaders may not be as high in status, they may be better able to tailor messages to local contexts and focus on community-specific issues.

In other contexts, trusted secular authorities may be more effective messengers among populations that are less religious or in which political divides are not religious in nature. A significant body of work suggests that lay authority figures of all kinds can be effective in changing minds and shifting norms (27, 29, 56), particularly those who are trusted and who are considered to be experts in a given domain (28, 57). More research is needed into whether domestic secular leaders are effective in promoting peace in conflict and postconflict settings, which kinds of secular leaders are most influential, and whether levels of trust or other characteristics condition this influence.

Third, our intervention does not permit us to disentangle whether our effects are caused by the religious content itself or the influence of a religious leader; we combine the two in the radio programs. This mimics how respondents often experience religion in the real world as a bundled treatment. Exposure to religious text on its own has been found to be very persuasive in influencing individual attitudes, particularly among highly religious populations (44) and when delivered by religious authorities (45). Future research should build on these experimental approaches and include exhaustive treatment conditions that both isolate the religious content of the message from the religious credentials of the messenger as well as combine the two to explore which elements might be causing the kind of effects we observe in this research.

Our results show that elevating the voices of leaders who promote reconciliation can be effective, even in the wake of violent conflict. Based on these results, we believe this can be a scalable and cost-effective intervention to foster positive social change. Messages from trusted authorities can reach hundreds or even tens of thousands of people at once through in-person speeches, broadcast media, and social media. For example, leaders can go on the radio or television and discuss their views in interviews or talk shows. In contexts in which Internet usage is common, they can spread written messages and short video clips on social media sites such as Facebook. Leaders can also work collaboratively with governments, nonprofits, and religious organizations to launch coordinated campaigns in which a number of local and national leaders all spread similar messages at the same time. We encourage scholars and practitioners to creatively build on our intervention and findings to more fully explore how trusted authorities can promote reconciliation and positive social change.

## Data Availability

Anonymized data have been deposited in OSF (DOI: 10.17605/OSF.IO/PU5YR).

## Footnotes

*We exclude from the main analyses a small number of Christian respondents (*n* = 90), who were randomized separately and whose treatment differed from the Muslims in the study (they received a message on reintegration from a Catholic bishop). Results do not change with their inclusion; we report the main analyses with Christians included in *SI Appendix*.

^†^The results do not differ substantively when analyzed using the difference-in-means estimator.

## References

[1] K. Koser, IDPs, Refugees, and Violent Extremism: From Victims to Vectors of Change (Brookings Institution, Washington, DC, 2020).

[2] Institute for Economics & Peace, Global Terrorism Index 2020: Measuring the Impact of Terrorism, Sydney, November 2020. https://www.visionofhumanity.org/maps/global-terrorism-index/#/. Accessed date 10 July 2021.

[3] S. Atran, Genesis of suicide terrorism. Science 299, 1534–1539 (2003). 12624256 10.1126/science.1078854

[4] W. B. Swann Jr, A. Gómez, D. C. Seyle, J. F. Morales, C. Huici, Identity fusion: The interplay of personal and social identities in extreme group behavior. J. Pers. Soc. Psychol. 96, 995–1011 (2009). 19379032 10.1037/a0013668

[5] A. W. Kruglanski ., The psychology of radicalization and deradicalization: How significance quest impacts violent extremism. Polit. Psychol. 35, 69–93 (2014).

[6] M. Sageman, Understanding Terror Networks (University of Pennsylvania Press, 2004).

[7] J. A. Carter, S. Maher, P. R. Neumann, #Greenbirds: Measuring Importance and Influence in Syrian Foreign Fighter Networks (International Centre for the Study of Radicalisation, 2014).

[8] E. Benmelech, E. F. Klor, What explains the flow of foreign fighters to ISIS? Terror. Polit. Violence 32, 1458–1481 (2020).

[9] H. Whitehouse, Dying for the group: Towards a general theory of extreme self-sacrifice. Behav. Brain Sci. 41, e192 (2018). 29409552 10.1017/S0140525X18000249

[10] E. Pokalova, Driving factors behind foreign fighters in Syria and Iraq. Stud. Confl. Terror. 42, 798–818 (2019).

[11] T. Mitts, From isolation to radicalization: Anti-Muslim hostility and support for ISIS in the West. Am. Polit. Sci. Rev. 113, 173–194 (2019).

[12] K. Kao, M. R. Revkin, Retribution or reconciliation? Post-conflict attitudes toward enemy collaborators. Am. J. Pol. Sci., in press.

[13] G. Holmer, A. Shtuni, Returning Foreign Fighters and the Reintegration Imperative (US Institute of Peace, 2017).

[14] M. Revkin, After the Islamic State: Balancing Accountability and Reconciliation in Iraq (United Nations University Centre for Policy Research, 2018).

[15] A. B. Bukarti, R. Bryson, Dealing with Boko Haram Defectors in the Lake Chad Basin: Lessons from Nigeria (Tony Blair Institute for Global Change, 2019).

[16] R. Wright, Despite Trump’s Guantánamo threats, Americans who joined ISIS are quietly returning home. The New Yorker, 11 June 2019. https://www.newyorker.com/news/news-desk/americas-isis-members-are-coming-home. Accessed 20 July 2021.

[17] A. Hoffman, M. Furlan, Challenges Posed by Returning Foreign Fighters (George Washington University Program on Extremism, 2020).

[18] J. Annan, C. Blattman, D. Mazurana, K. Carlson, Civil war, reintegration, and gender in Northern Uganda. J. Confl. Resolut. 55, 877–908 (2011).

[19] M. J. Gilligan, E. N. Mvukiyehe, C. Samii, Reintegrating rebels into civilian life: Quasi-experimental evidence from Burundi. J. Confl. Resolut. 57, 598–626 (2013).

[20] International Crisis Group, “Averting an ISIS resurgence in Iraq and Syria” (*Middle East & North Africa Rep. No. 207*, International Crisis Group, 2019).

[21] Human Rights Watch, Scant help to bring home ISIS members’ children. Human Rights Watch News, 12 February 2019. https://www.hrw.org/news/2019/02/12/tunisia-scant-help-bring-home-isis-members-children#. Accessed 20 July 2021.

[22] R. Littman, E. L. Paluck, The cycle of violence: Understanding individual participation in collective violence. Polit. Psychol. 36, 79–99 (2015).

[23] L. Grip, J. Kotajoki, Deradicalisation, disengagement, rehabilitation and reintegration of violent extremists in conflict-affected contexts: A systematic literature review. Confl. Secur. Dev. 19, 371–402 (2019).

[24] M. Wessells, Child Soldiers: From Violence to Protection (Harvard University Press, 2006).

[25] R. B. Cialdini, M. R. Trost, “Social influence: Social norms, conformity and compliance” in The Handbook of Social Psychology (McGraw-Hill, ed. 4, 1998), vol. 1–2, pp. 151–192.

[26] M. Sherif, The Psychology of Social Norms (Harper, Oxford, England, 1936).

[27] J. R. Zaller, The Nature and Origins of Mass Opinion (Cambridge University Press, 1992).

[28] J. N. Druckman, A. Lupia, Preference formation. Annu. Rev. Polit. Sci. 3, 1–24 (2000).

[29] G. S. Lenz, Follow the Leader?: How Voters Respond to Politicians’ Policies and Performance (University of Chicago Press, 2012).

[30] M. S. Y. Chwe, Rational Ritual: Culture, Coordination, and Common Knowledge (Princeton University Press, 2013).

[31] M. A. Hogg, “Influence and leadership” inThe Handbook of Social Psychology (JohnWiley & Sons, Inc., ed. 5, 2010), vol. 2, pp. 1166–1207.

[32] M. E. Tankard, E. L. Paluck, Norm perception as a vehicle for social change. Soc. Issues Policy Rev. 10, 181–211 (2016).

[33] T. C. Helmus, E. York, P. Chalk, Promoting Online Voices for Countering Violent Extremism (RAND, 2013).

[34] A. Grossman, W. G. Nomikos, N. Siddiqui, Can appeals for peace promote tolerance and mitigate support for extremism? Evidence from an experiment with adolescents in Burkina Faso. Open Science Framework [Preprint] (2021). 10.31219/osf.io/49na5 (Accessed 27 September 2021).

[35] J. Krause, Non-violence and civilian agency in communal war: Evidence from Jos, Nigeria. Afr. Aff. (Lond.) 116, 261–283 (2017).

[36] R. A. Blair, International intervention and the rule of law after civil war: Evidence from Liberia. Int. Organ. 73, 365–398 (2019).

[37] W. G. Nomikos, Peacekeeping and the enforcement of intergroup cooperation: Evidence from Mali. J. Polit., 10.1086/715246 in press.

[38] United Nations High Commissioner for Refugees, Operational Data Portal Regional Response - Nigeria Situation: Nigerian Refugees in Chad, Cameroon and Niger (UNHCR, 2019).

[39] V. Felbab-Brown, “In Nigeria, We Don”t Want Them Back:’ Amnesty, Defectors’ Programs, Leniency Measures, Informal Reconciliation, and Punitive Responses to Boko Haram (United Nations University Centre for Policy Research, 2018).

[40] Pew Research Center, The Global Religious Landscape: A Report on the Size and Distribution of the World’s Major Religious Groups as of 2010 (Pew Research Center, 2012).

[41] P. A. Djupe, C. P. Gilbert, The Prophetic Pulpit: Clergy, Churches, and Communities in American Politics (Rowman & Littlefield, 2003).

[42] G. H. McClendon, R. B. Riedl, From Pews to Politics: Religious Sermons and Political Participation in Africa (Cambridge University Press, 2019).

[43] G. H. McClendon, R. B. Riedl, Using sermons to study religions’ influence on political behavior. Comp. Polit. Stud. 54, 779–822 (2021).

[44] T. Masoud, A. Jamal, E. Nugent, Using the Qur’ ¯an to empower Arab women? Theory and experimental evidence from Egypt. Comp. Polit. Stud. 49, 1555–1598 (2016).

[45] L. N. Condra, M. Isaqzadeh, S. Linardi, Clerics and scriptures: Experimentally disentangling the influence of religious authority in Afghanistan. Br. J. Polit. Sci. 49, 401–419 (2019).

[46] G. Blair, J. Cooper, A. Coppock, M. Humphreys, Declaring and diagnosing research designs. Am. Polit. Sci. Rev. 113, 838–859 (2019). 32855557 10.1017/s0003055419000194 PMC7449569

[47] W. Lin, Agnostic notes on regression adjustments to experimental data: Reexamining Freedman’s critique. Ann. Appl. Stat. 7, 295–318 (2013).

[48] E. L. Paluck, Reducing intergroup prejudice and conflict using the media: A field experiment in Rwanda. J. Pers. Soc. Psychol. 96, 574–587 (2009). 19254104 10.1037/a0011989

[49] T. C. Davenport ., The enduring effects of social pressure: Tracking campaign experiments over a series of elections. Polit. Behav. 32, 423–430 (2010).

[50] S. Murrar, M. Brauer, Entertainment-education effectively reduces prejudice. Group Process. Intergroup Relat. 21, 1053–1077 (2018).

[51] G. Blair, R. Littman, E. L. Paluck, Motivating the adoption of new community-minded behaviors: An empirical test in Nigeria. Sci. Adv. 5, eaau5175 (2019). 30891494 10.1126/sciadv.aau5175 PMC6415959

[52] J. Schwedler, Can Islamists become moderates? Rethinking the inclusion-moderation hypothesis. World Polit. 63, 347–376 (2011).

[53] D. C. Leege, L. A. Kellstedt, Rediscovering the Religious Factor in American Politics (ME Sharpe, 1993).

[54] J. L. Guth, J. C. Green, L. A. Kellstedt, C. E. Smidt, Faith and the environment: Religious beliefs and attitudes on environmental policy. Am. J. Pol. Sci. 39, 364–382 (1995).

[55] G. H. McClendon, R. B. Riedl, Religion as a stimulant of political participation: Experimental evidence from Nairobi, Kenya. J. Polit. 77, 1045–1057 (2015).

[56] T. Brader, J. A. Tucker, D. Duell, Which parties can lead opinion? Experimental evidence on partisan cue taking in multiparty democracies. Comp. Polit. Stud. 46, 1485–1517 (2013).

[57] M. Gilens, N. Murakawa, Elite cues and political decision-making. Res. Micropolitics 6, 15–49 (2002).

